# Developing risk stratification strategies and biomarkers for recurrent hepatocellular carcinoma

**DOI:** 10.1002/ctm2.70410

**Published:** 2025-07-31

**Authors:** Jia‐Hao Law, Huilin Shao, Ramanuj DasGupta, Daniel Q. Huang

**Affiliations:** ^1^ Division of Hepatobiliary & Pancreatic Surgery Department of Surgery National University Hospital Singapore; ^2^ Institute for Health Innovation and Technology National University of Singapore Singapore; ^3^ Department of Biomedical Engineering College of Design and Engineering National University of Singapore Singapore; ^4^ Department of Surgery, Yong Loo Lin School of Medicine National University of Singapore Singapore; ^5^ Institute of Molecular and Cell Biology Agency for Science Technology and Research Singapore; ^6^ Laboratory of Precision Medicine and Cancer Evolution Genome Institute of Singapore Agency for Science, Technology and Research (A^*^STAR) Singapore Singapore; ^7^ Division of Gastroenterology and Hepatology Department of Medicine National University Hospital Singapore; ^8^ National University Centre for Organ Transplantation National University Health System Singapore

**Keywords:** HCC biomarkers, hepatocellular carcinoma recurrence, risk stratification HCC

## Abstract

Hepatocellular carcinoma (HCC) remains a leading cause of cancer‐related mortality, with high rates of post‐resection recurrence posing significant clinical challenges. Early recurrence is largely driven by aggressive tumor biology, while late recurrence reflects de novo carcinogenesis in a cirrhotic liver. Traditional clinical and pathological predictors are insufficient for accurately identifying high‐risk patients. Emerging translational advances including genomic, transcriptomic, proteomic, and metabolomic biomarkers; liquid biopsy techniques; artificial intelligence (AI)‐driven histological and radiomic analyses offer new avenues to refine recurrence risk stratification and guide perioperative therapy. Simultaneously, the shifting etiological landscape from viral hepatitis to metabolic dysfunction‐associated steatohepatitis (MASH) and alcohol‐related liver disease underscores the need for tailored surveillance and preventive strategies. Advanced technologies such as single‐cell and spatial transcriptomics provide unprecedented insights into fibrosis progression and tumor evolution. Integrating these approaches may enable personalized surveillance protocols and therapeutic interventions, optimizing outcomes for HCC patients and reducing unnecessary resource utilization.

Hepatocellular carcinoma (HCC) is the third leading cause of cancer death worldwide. Although surgical resection is a primary treatment option for HCC, the five‐year recurrence‐free survival rate is only 35%.^1^ HCC recurrence is characterised by a bimodal pattern: early recurrences, occurring within the first two years, are primarily due to residual microscopic disease or occult intrahepatic metastasis, while late recurrences are driven by de novo carcinogenesis in a cirrhotic liver.

Early recurrences of HCC are primarily driven by tumours exhibiting aggressive biological behaviour. Conventional predictors of recurrence, such as tumour size, number, differentiation, lymphovascular invasion, and microvascular invasion, while essential to current guidelines and studies, offer limited precision in identifying high‐risk patients.^2^ These parameters fail to capture the complex and heterogeneous nature of HCC recurrence. This limitation underscores the urgent need for more sophisticated and accurate predictive tools. The development of advanced biomarkers and predictive models is particularly crucial in light of emerging evidence supporting the efficacy of adjuvant therapies for HCC patients. A phase III trial of atezolizumab plus bevacizumab in patients with high‐recurrence risk HCC—defined by composite criteria including tumour size, number, microvascular invasion, segmental portal vein invasion, or poor differentiation—met its primary endpoint of recurrence‐free survival, although it did not significantly impact overall survival.^3^ Enhanced predictive capabilities may enable better selection of patients with aggressive tumour biology who are more likely to benefit from perioperative treatments, in both neoadjuvant and adjuvant settings.

In recent years, there has been a growing interest in identifying and validating biomarkers that could provide deeper insights into the likelihood of recurrence and the aggressiveness of HCC. Biomarkers derived from genomic, transcriptomic, proteomic, and metabolomic studies hold promise for discerning tumour biology, predicting recurrence, and tailoring treatment strategies. For instance, genomic biomarkers such as mutations in the TERT promoter and CTNNB1 gene have been associated with HCC recurrence.^4^ Transcriptomic analyses have identified specific mRNA expression profiles that correlate with poor prognosis, such as elevated levels of microRNAs miR‐21 and miR‐221.^5^ Proteomic studies have highlighted proteins like des‐gamma‐carboxy prothrombin (DCP) as potential indicators of recurrence.^6^ Metabolomic profiling has revealed alterations in metabolites like bile acids and phospholipids that are linked to HCC progression.^7^

Despite the potential of these biomarkers, their clinical application has been limited related to the lack of large, phase III studies. For example, while liquid biopsy techniques have shown promise in detecting circulating tumour DNA (ctDNA) and circulating tumour cells (CTCs) associated with HCC,^8^ these methods require further validation through large‐scale clinical trials to establish their reliability and clinical utility. The challenge lies in the need for robust, large‐scale studies to validate these biomarkers and integrate them into clinical practice. Developing standardised protocols for biomarker testing and interpretation is also crucial.

Advances in technologies such as next‐generation sequencing (NGS) and liquid biopsy are paving the way for more precise and personalised predictive models.^9^ NGS allows for comprehensive genomic profiling of tumours, identifying mutations and alterations that may predict recurrence risk. Additionally, the application of machine learning and artificial intelligence (AI) to histological slides has shown potential in identifying novel histological traits that correlate with HCC outcomes.^10^ AI algorithms can analyse vast amounts of data from digital pathology images, uncovering patterns that may be missed by human observers. Radiomics, which involves extracting quantitative features from medical imaging, is another emerging field that could enhance predictive accuracy. Radiomic analyses of magnetic resonance imaging (MRI) and computed tomography (CT) images have been used to identify imaging biomarkers associated with tumour heterogeneity and recurrence.^11^ Integrating radiomic features with clinical and genomic data could lead to comprehensive predictive models that inform personalised surveillance strategies and treatment plans.

Delayed recurrences of HCC are predominantly associated with underlying field changes in the native liver. These recurrences are often the result of de novo tumour formation in a chronically diseased liver environment. The prevalence of hepatitis B virus (HBV) and hepatitis C virus (HCV) is declining due to the advent of increasingly effective and affordable antiviral treatments, leading to significant improvements in RFS among patients with viral hepatitis‐associated HCC.^12^ These antiviral therapies not only achieve viral suppression but also attenuate liver inflammation and fibrosis, thereby mitigating the risk of new tumour development. Furthermore, overall survival is markedly improved in patients who persist with HBV therapy post‐resection. Continuous antiviral therapy plays a crucial role in maintaining viral suppression, reducing the progression of liver damage, and preventing further hepatocarcinogenesis.

As the incidence of viral hepatitis‐associated HCC declines, there is a growing focus on HCC etiologies linked to metabolic dysfunction‐associated steatohepatitis (MASH) and alcohol‐related liver disease (ALD).^13^ These conditions are emerging as significant contributors to liver cirrhosis and subsequent HCC, driven by the global rise in obesity, metabolic syndrome, and alcohol consumption.^14^ This shift necessitates the development of specialised surveillance and therapeutic strategies tailored to these etiologies. A critical area of research is the reversal of cirrhosis or fibrosis associated with metabolic dysfunction‐associated steatotic liver disease (MASLD) and MASH. Liver fibrosis and cirrhosis are pivotal risk factors for HCC, representing advanced stages of liver disease with a high potential for malignant transformation. Next‐generation single‐cell and spatial technologies are revolutionising the study of molecular mechanisms driving the progression of MASH/MAFLD‐related cirrhosis to HCC. These advanced technologies offer unparalleled resolution and precision in the analysis of cellular heterogeneity, tissue architecture, and microenvironmental interactions that have been shown to play crucial roles in HCC development.^15^, ^16^ scRNA‐seq enables the identification of rare founder cell populations, such as cancer stem cells, specific immune cell subsets, and stromal cells, including remodeled endothelial cells and fibroblasts, which play critical roles in fibrosis and tumorigenesis. By profiling gene expression at the individual cell level, single‐cell RNA‐sequencing helps uncover “cell state”‐associated biomarkers that define the cellular origins of fibrosis and cirrhosis and identify molecular drivers of their transition to HCC. Additionally, spatial transcriptomic technologies visualise interactions between different cell types and the extracellular matrix, essential for identifying microenvironmental factors contributing to fibrosis and cirrhosis and pinpointing regions of the liver prone to malignant transformation. Current research efforts are exploring antifibrotic agents, lifestyle modifications, and novel therapeutic targets aimed at halting or reversing liver fibrosis.^17^ Elucidating the molecular and genetic drivers of MASH and ALD‐related HCC is imperative, as identifying specific biomarkers for these conditions could significantly enhance early detection and facilitate personalised treatment approaches. The development of potent antifibrotic therapies and effective lifestyle interventions has the potential to substantially reduce HCC incidence within these high‐risk cohorts. Integrating these advanced technologies to interrogate relevant clinical samples can provide high‐resolution, context‐rich data, essential for discovering new biomarkers and therapeutic targets, ultimately leading to more effective interventions to prevent HCC recurrence and improve patient prognosis.

As HCC patients experience longer survival, conventional surveillance strategies warrant reevaluation. Routine 3–12 monthly contrasted MRI or CT scans, depending on the guidelines followed, may not be necessary for all patients, given their varying recurrence profiles. Instead, surveillance intervals could be tailored based on recurrence risks predicted by advanced predictive models. Additionally, novel modalities can be integrated into clinical care to enhance efficiency and reduce costs. Emerging evidence suggests that abbreviated MRI (aMRI) has comparable sensitivity to full‐sequence contrasted MRI but without the risks associated with contrast agents and potentially offers a more cost‐effective option.^18^, ^19^ Blood‐based biomarkers such as PIVKA‐II and ctDNA to detect minimal residual disease can also be incorporated into surveillance protocols,^20^, ^21^ either as standalone measures or in combination with imaging, to maintain efficacy while reducing costs.

Novel translational research and optimising surveillance strategies have the potential to improve patient outcomes and improve resource utilisation for patients undergoing resection for HCC. A personalised approach to HCC management, incorporating advanced biomarkers to discern tumour biology before resection, innovative imaging surveillance techniques, and predictive biomarkers for systemic therapies, holds promise to transform the surveillance paradigm and treatment landscape.

## AUTHOR CONTRIBUTIONS

Jia‐Hao Law and Daniel Q. Huang conceived and drafted the manuscript, as well as drew the figures. Huilin Shao and Ramanuj DasGupta provided valuable discussion and revised the manuscript. All authors have read and approved the article.

## CONFLICT OF INTEREST STATEMENT

Daniel Q. Huang has served as an advisory board member for Gilead and Roche.

## FUNDING INFORMATION

Daniel Q. Huang receives funding support from the Singapore Ministry of Health's National Medical Research Council (MOH‐001370) and the NUHS Clinician Scientist Program (NCSP2.0/2023/NUHS/DH).

## ETHICS STATEMENT

The authors have nothing to report.
